# *Escherichia coli* Lacking RpoS Are Rare in Natural Populations of Non-Pathogens

**DOI:** 10.1534/g3.112.003855

**Published:** 2012-11-01

**Authors:** Emily Snyder, David M. Gordon, Daniel M. Stoebel

**Affiliations:** * Department of Biology, Harvey Mudd College, Claremont California 91711; † Research School of Biology, Australian National University, Canberra, Australian Capital Territory 0200, Australia

**Keywords:** SPANC, population genetics, variation, transcription factor

## Abstract

The alternative sigma factor RpoS controls a large regulon that allows *E. coli* to respond to a variety of stresses. Mutations in *rpoS* can increase rates of nutrient acquisition at the cost of a decrease in stress resistance. These kinds of mutations evolve rapidly under certain laboratory conditions where nutrient acquisition is especially challenging. The frequency of strains lacking RpoS in natural populations of *E. coli* is less clear. Such strains have been found at frequencies over 20% in some collections of wild isolates. However, laboratory handling can select for RpoS-null strains and may have affected some of these strain collections. Other studies have included an unknown diversity of strains or only used a phenotypic proxy as a measure of RpoS levels. We directly measured RpoS levels in a collection of *E. coli* that includes the full diversity of the species and that was handled in a manner to minimize the potential for laboratory evolution. We found that only 2% of strains produce no functional RpoS. Comparison of these strains in multiple labs shows that these *rpoS* mutations occurred in the laboratory. Earlier studies reporting much higher levels of RpoS polymorphism may reflect the storage history of the strains in laboratories rather than true frequency of such strains in natural populations.

Free-living bacteria respond to changes in their environment by altering their patterns of gene expression. In *Escherichia coli* and its relatives, the alternative sigma factor RpoS coordinates responses to stresses like low temperature, high osmolarity, and starvation (Battesti *et al.* 2011; Hengge 2011). RpoS, like all sigma factors, acts by recruiting core RNA polymerase to a set of promoters whose DNA sequence it recognizes (Typas *et al.* 2007). When RpoS recruits RNA polymerase to its target promoters, transcription from promoters that depend on other sigma factors is reduced. This phenomenon, called sigma factor competition (Farewell *et al.* 1998; Nyström 2004), is thought to occur because there is little free core RNA polymerase in the cell at a given time, so each act of transcribing a RpoS-dependent promoter deprives another promoter of the chance for transcription.

Sigma factor competition implies that bacteria with relatively high levels of transcription of genes conferring resistance to physical stresses like low temperature or high osmolarity are less able to transcribe genes that allow them to metabolize a variety of nutrients (Ferenci 2005). This trade-off suggests that strains of *E. coli* cannot evolve to become both better able to resist stresses and better able to acquire nutrients. Rather, strains can improve one of these two traits only at the cost of the other by altering the level of RpoS they produce.

One of the critical observations supporting this hypothesis is that the level of RpoS is a frequent target for evolutionary change in the laboratory. When *E. coli* is cultured in long-term stationary phase, mutations that severely attenuate levels of RpoS activity provide a fitness benefit (Zambrano *et al.* 1993; Farrell and Finkel 2003; Finkel 2006). Similarly, growth in chemostats rapidly selects for strains with null alleles of *rpoS* (Notley-Mcrobb *et al.* 2002; King *et al.* 2006). In addition, microbiologists have unwittingly performed experimental evolution on RpoS levels in the course of their routine lab work. For example, the storage of *E. coli* and its close relative *Salmonella enterica* as stabs selects for partial or complete loss of function mutations in *rpoS* (Sutton *et al.* 2000; Spira *et al.* 2011), as does the routine practice of shipping strains on glycerolized filter disks (Spira *et al.* 2011).

Complementing these studies of the selective benefit of mutations in *rpoS* in laboratory environments, several studies have documented variation in levels of RpoS protein in collections of naturally occurring *E. coli* and its relatives. In a collection of enterohemorrhagic *E. coli*, 24% of strains were demonstrated to have non-functional RpoS (Bhagwat *et al.* 2005). Likewise, 37% of clinical *S. enterica* serotype Typhi isolates were found to have non-functional *rpoS* alleles, although the same study noted that no isolates with null *rpoS* alleles were found among 75 clinical *S. enterica* serotype Tyhpimurium isolates (Robbe-Saule *et al.* 2003). Ferenci *et al.* (2011) found that 9 of 31 (29%) strains from the ECOR collection, which includes much of the diversity of *E. coli* (Ochman and Selander 1984), had no detectible RpoS protein, as judged by western blotting.

In this study, we provide an estimate of the frequency of *rpoS*-null alleles in a new collection of non-pathogenic *E. coli*. This collection includes the full known diversity of *E. coli*. Surprisingly, we find that strains lacking RpoS are rare in our collection.

## Materials and Methods

### Strains

The strains examined represent a subset of isolates collected from humans, other vertebrates, and environmental sources in Australia. The procedures used to isolate these strains have been previously described (Gordon and Fitzgibbon 1999; Gordon and Cowling 2003; Gordon *et al.* 2005). Once isolated as pure cultures, strains were stored at −80°. Strains were characterized using multi-locus sequence typing and were assigned to one of the main phylogroups of *E. coli* or its close relatives (Gordon *et al.* 2008; Walk *et al.* 2009; Tenaillon *et al.* 2010).

Strains were shipped from the lab of D. Gordon to the STEC center at Michigan State University as agar stabs, where they were stored as glycerolized cultures at −80°. Strains were sent to the Stoebel lab as agar stabs, where they were again stored as glycerolized cultures at −80°.

### Western blotting

Western blotting (Gallagher *et al.* 2011) was used to determine the amount of RpoS protein present in each strain. Strains were grown in 3 ml cultures of LB (5 g yeast extract, 10 g tryptone, 10 g NaCl per liter water) for 20 hr. To harvest protein from these stationary phase cultures, 100 μL of culture was removed and centrifuged, then resuspended in 200 µL 1× Laemmli sample loading buffer (Sigma) and heated at 100° for 5 min. A 10 µL aliquot was run through SDS-PAGE (10% acrylamide) and electrophoreticaly transferred to a Hybond-LPF PVDF membrane (GE Healthcare) at 100 V at 4° for 2 hr or 40 V at 4° overnight. After submersion in blocking solution (5% nonfat skim milk in TBST) overnight, the membrane was probed with primary antibodies for RpoS or RpoA (both from NeoClone) (diluted 1:10,000) for 1 hr. After washing in TBST, the membrane was probed with HRP-conjugated goat anti-mouse secondary antibodies (Jackson ImmunoResearch) (diluted 1:5,000) for 1 hr. The Amersham ECL Plus Western Blotting Detection System (GE Healthcare) was used for detection on a Typhoon imager (GE Healthcare). At least two independent protein samples were prepared for each strain. Each experiment included positive [CF7968 (Brown *et al.* 2002)] and Δ*rpoS* [DMS1688 (Stoebel *et al.* 2009)] control strains.

### PCR, DNA sequencing, and analysis

Genomic DNA was purified using the Gentra Puregene cell kit (Qiagen) and used for PCR. The *rpoS* gene was amplified with primers rpoS.ext.seq2+ (5′-CCCGCTGCGTTATTTGCCGC-3′) and rpoS.ext.seq2− (5′-AGCCTCGCTTGAGACTGGCCT-3′). Amplification was performed with HotStarTaq Plus (Qiagen) using the manufacturers recommendations. The resulting PCR products were sequenced with those primers and primers rpoS.int.seq+ (5′-GCCAGACGATTGAACGGGCGA-3′) and rpoS.int.seq− (5′-TGGCGAATCCACCAGGTTGCG-3′). The *rpoS* gene sequences of all but four strains used in this study (B1167, H260, TA155, and TA445) were downloaded from the *Escherichia coli* Antibiotic Resistance Sequencing Project, Broad Institute of Harvard and MIT (http://www.broadinstitute.org/) on June 12, 2012.

## Results

We studied RpoS production in a collection of 96 strains that included representatives of all major lineages of *E. coli* sensu stricto (A, B1, B2, D, E, F, and *Shigella*) as well as the other named species of *Escherichia* and recently discovered cryptic lineages of *Escherichia* (Walk *et al.* 2009). One strain could not be assigned to any clonal group, but its DNA sequences cluster within the phylogeny of *E. coli*. A summary of the distribution of the strains is given in Table 1, with full details shown in supporting information, Table S1.

**Table 1:** Phylogenetic distribution of strains of *E. coli* examined in this study

Phylogroup	Number of Strains
A	23
B1	16
B2	25
CI	4
CIII	1
CIV	1
CV	2
D	14
E	4
*Escherichia albertii*	2
*Escherichia fergusonii*	1
“*Shigella*”*^a^*	1
Unassigned*^b^*	1

All major *E. coli* phylogroups as well as all major *Escherichia* lineages are represented.

a A non-pathogenic member of the Shigella clonal groups.

b This strain could not be assigned to any named phylogroup.

We measured RpoS levels directly by Western blotting. In our collection of 96 strains, we discovered only 2 that fail to produce RpoS: strain M114 and strain M646, which are both from phylogroup D. As a control, we also probed for the presence of RpoA, a subunit of core RNA polymerase that is presumably essential for viability in all strains. RpoA was detected for all protein samples from all strains, indicating that all samples were properly prepared. Thus, we find that only 2 of 96 (2%) of strains of *E. coli* and close relatives are RpoS negative. This is significantly lower [*P* < 0.0001, Chi-square test with a Monte Carlo-derived *P*-value (R Development Core Team 2009)] than the previous finding of Ferenci *et al.* (2011), who found 29% of strains in the ECOR collection did not produce RpoS.

We PCR-amplified and sequenced the *rpoS* gene from strains M114 and M646 to identify the mutation causing the RpoS-null phenotype. Strain M114 contains an insertion of TA between bases 391 and 392 of the coding sequence. This frameshift mutation results in a premature stop codon and a polypeptide of 131 rather than 331 amino acids. Strain M646 contains a deletion of bases 614–623, a frameshift resulting in a polypeptide of 218 amino acids.

Lab-specific alleles of *rpoS* can easily arise during storage and transport (Ferenci *et al.* 2011). To see whether the *rpoS* frameshift mutations in M114 and M646 were specific to our lab, we compared the sequences from the strains in our lab with the *rpoS* sequence of these strains determined at the Broad Institute. The Broad Institute sequences did not contain the frameshift mutations found in our versions of these same strains. In fact, none of the strains sequenced by the Broad Institute showed frameshift or nonsense mutations in *rpoS*. We infer that the only strains that lack RpoS have evolved this phenotype in the laboratory.

## Discussion

Polymorphism in RpoS has been reported to be widespread in both natural isolates and laboratory populations of *E. coli*. Among non-pathogenic *E. coli*, only one previous study has surveyed RpoS levels in a collection known to include the breadth of *E. coli* diversity. In that study, Ferenci *et al.* (2011) found that 9 of 31 (29%) strains in the ECOR collection did not produce RpoS. In contrast, we found that only 2 of 96 strains (2%) in our collection did not produce RpoS. Though these two strains had frameshift mutations, these mutations were specific to our lab and not present in the genomes of these same strains in another lab.

At least three factors might result in differences between studies in the estimates of strains lacking RpoS. First, studies can differ in how the RpoS status of strains was measured. Some studies have relied purely on phenotypic measures of the RpoS status of strains [*e.g.* White *et al.* (2011)]. This approach includes in the tally of RpoS-negative strains those strains with mutations in other genes responsible for the phenotype. It also counts as RpoS-positive those strains that lack RpoS but whose regulatory network differs such that it does not influence the phenotype of interest. Both kinds of strains have been documented (Robbe-Saule *et al.* 2003; Bhagwat *et al.* 2005, 2006), so this is not the best way to measure the frequency of RpoS-null strains.

Western blotting is a more direct way than phenotypic proxies to measure RpoS levels. However, Western blotting still classifies as RpoS-positive strains that produce full-length but non-functional RpoS proteins. Future work with this collection could test the ability of *rpoS* genes to complement a Δ*rpoS* mutation in *E. coli* K-12. The lack of direct evidence about the functionality of RpoS in our strains is not an explanation for the difference between this study and that of Ferenci *et al.* (2011), as both studies used Western blotting to directly measure RpoS levels.

A second reason that studies might differ is the diversity of the strains considered. *E. coli* is a very diverse species found in a wide variety of hosts and non-host environments. Some studies have explicitly examined specific lineages of *E. coli* (Bhagwat *et al.* 2005, 2006). Others have examined *E. coli* from specific environments without explicit regard to lineage (Chiang *et al.* 2011). Finally, this work and that of Ferenci *et al.* (2011) examined strain collections that were assembled to include the diversity of *E. coli*. As the impact of mutations in *rpoS* are known to have different effects in different lineages (Ferenci *et al.* 2011), studies that differ in the strains examined may come to different conclusions about the frequency of the RpoS-null alleles. This reason is unlikely to explain the difference between our study and Ferenci *et al.* (2011), as both collections include the diversity of *E. coli*.

Finally, studies may differ in the storage history of the strains used. Storage in stab cultures and transport as glycerolized cultures on filter disks is known to select for RpoS-null strains (Sutton *et al.* 2000; Spira *et al.* 2011). We believe that this is the best explanation for the difference between our study and that of Ferenci *et al.* (2011). The strains of the ECOR collection were stored for variable periods as stabs or slants before the collection was assembled and distributed (D. Dykhuizen and H. Ochman, personal communication). In contrast, the strains used in this study were frozen at −80° as quickly as possible. We propose that the relatively high frequency of RpoS-null strains in the ECOR collection reflects their storage history and not the frequency of null RpoS alleles in natural populations of *E. coli*. Our finding that the strains in our collection bearing null *rpoS* alleles are specific to our lab is consistent with the interpretation that such strains are a product of selection during shipping rather than commonly occurring in nature.

Although working with a smaller strain collection, the work of Levert *et al.* (2010) is consistent with our study. Those authors examined the heterogenetiy of extraintestinal *E. coli* isolates from patients. Isolates from single patients were variable for many traits commonly taken as a marker of variation in RpoS levels (breadth of carbon source utilization, motility, H_2_O_2_ and acid sensitivity, glycogen production, and the red dry and rough morphotype). In spite of this phenotypic variation, no mutations in *rpoS* or its promoter were found, and RpoS was detected by Western blot from all isolates.

Other studies measuring RpoS levels are subject to one or more of the above caveats. For example, Chiang *et al.* (2011) discovered six strains lacking RpoS among 2040 environmental isolates of *E. coli*. They first screened the collection for strains with phenotypes similar to an *E. coli* K-12 strain lacking RpoS, meaning that they missed any strains that lack RpoS but did not match their phenotypic expectation. As such, 0.3% should be taken as a lower bound on the frequency of strains lacking RpoS in environmental *E. coli*. Nonetheless, our 2% estimate is not significantly different (*P* = 0.047, Chi-square test with a Monte Carlo-derived *P*-value; the Bonferroni-corrected significance level is 0.025) from the 0.3% estimate obtained by Chiang *et al.* (2011).

In conclusion, we argue that null *rpoS* alleles are rare across the full diversity of non-pathogenic *E. coli*. This does not necessarily imply that null *rpoS* alleles are similarly rare within specific lineages or in specific environments. It is possible that certain pathogenic lineages of *E. coli* might have relatively high levels of null alleles. Our work does, however, urge caution in interpreting results of surveys of *rpoS* polymorphism unless RpoS protein levels have been measured directly, the scope of the collection is considered, and the isolation and storage conditions are known to have minimized the chance for laboratory selection.

## Supplementary Material

Supporting Information

## References

[bib1] Battesti A. Majdalani N. Gottesman S., 2011 The RpoS-mediated general stress response in *Escherichia coli*. Annu. Rev. Microbiol. 2011: 189–213 21639793 10.1146/annurev-micro-090110-102946 PMC7356644

[bib2] Bhagwat A. A. Chan L. Han R. Tan J. Kothary M., 2005 Characterization of enterohemorrhagic *Escherichia coli* strains based on acid resistance phenotypes. Infect. Immun. 73: 4993–5003 16041014 10.1128/IAI.73.8.4993-5003.2005 PMC1201262

[bib3] Bhagwat A. A. Tan J. Sharma M. Kothary M. Low S., 2006 Functional heterogeneity of RpoS in stress tolerance of enterohemorrhagic Escherichia coli strains. Appl. Environ. Microbiol. 72: 4978–4986 16820496 10.1128/AEM.02842-05 PMC1489321

[bib4] Brown L. Gentry D. Elliott T. Cashel M., 2002 DksA affects ppGpp induction of RpoS at a translational level. J. Bacteriol. 184: 4455–4465 12142416 10.1128/JB.184.16.4455-4465.2002 PMC135238

[bib5] Chiang S. M. Dong T. Edge T. A. Schellhorn H. E., 2011 Phenotypic diversity caused by differential RpoS activity among environmental Escherichia coli isolates. Appl. Environ. Microbiol. 77: 7915–7923 21948830 10.1128/AEM.05274-11 PMC3209002

[bib6] Farewell A. Kvint K. Nyström T., 1998 Negative regulation by RpoS: a case of sigma factor competition. Mol. Microbiol. 29: 1039–1051 9767572 10.1046/j.1365-2958.1998.00990.x

[bib7] Farrell M. J. Finkel S. E., 2003 The growth advantage in stationary-phase phenotype conferred by rpoS mutations is dependent on the pH and nutrient environment. J. Bacteriol. 185: 7044–7052 14645263 10.1128/JB.185.24.7044-7052.2003 PMC296246

[bib8] Ferenci T., 2005 Maintaining a healthy SPANC balance through regulatory and mutational adaptation. Mol. Microbiol. 57: 1–8 15948944 10.1111/j.1365-2958.2005.04649.x

[bib9] Ferenci T. Galbiati H. F. Betteridge T. Phan K. Spira B., 2011 The constancy of global regulation across a species: the concentrations of ppGpp and RpoS are strain-specific in Escherichia coli. BMC Microbiol. 11: 62 21439067 10.1186/1471-2180-11-62 PMC3074542

[bib10] Finkel S. E., 2006 Long-term survival during stationary phase: evolution and the GASP phenotype. Nat. Rev. Microbiol. 4: 113–120 16415927 10.1038/nrmicro1340

[bib11] Gallagher S. Winston S. E. Fuller S. A. Hurrell J. G. R., 2011 Immunoblotting and immunodetection, Unit 6.2 in *Current Protocols in Cell Biology*, edited by J. S. Bonifacino, M. Dasso, J. B. Harford, J. Lippincott-Schwartz, and K. M. Yamada. John Wiley & Sons, Inc., Hoboken, NJ

[bib12] Gordon D. M. Cowling A., 2003 The distribution and genetic structure of Escherichia coli in Australian vertebrates: host and geographic effects. Microbiology 149: 3575–3586 14663089 10.1099/mic.0.26486-0

[bib13] Gordon D. M. FitzGibbon F., 1999 The distribution of enteric bacteria from Australian mammals: host and geographical effects. Microbiology 145: 2663–2671 10537188 10.1099/00221287-145-10-2663

[bib14] Gordon D. M. Stern S. E. Collignon P. J., 2005 Influence of the age and sex of human hosts on the distribution of Escherichia coli ECOR groups and virulence traits. Microbiology 151: 15–23 15632421 10.1099/mic.0.27425-0

[bib15] Gordon D. M. Clermont O. Tolley H. Denamur E., 2008 Assigning Escherichia coli strains to phylogenetic groups: multi-locus sequence typing *vs.* the PCR triplex method. Environ. Microbiol. 10: 2484–2496 18518895 10.1111/j.1462-2920.2008.01669.x

[bib16] Hengge R., 2011 Stationary-phase gene regulation in Escherichia coli, Chapter 5.6.3 in *EcoSal—Escherichia coli and Salmonella: Cellular and Molecular Biology*, edited by A. Böck, R. Curtiss III, J. B. Kaper *et al*., ASM Press, Washington, DC. Available at: www.ecosal.org

[bib17] King T. Seeto S. Ferenci T., 2006 Genotype-by-environment interactions influencing the emergence of rpoS mutations in Escherichia coli populations. Genetics 172: 2071–2079 16489226 10.1534/genetics.105.053892 PMC1456365

[bib18] Levert M. Zamfir O. Clermont O. Bouvet O. Lespinats S., 2010 Molecular and evolutionary bases of within-patient genotypic and phenotypic diversity in Escherichia coli extraintestinal infections. PLoS Pathog. 6: e1001125 20941353 10.1371/journal.ppat.1001125 PMC2947995

[bib19] Notley-McRobb L. King T. Ferenci T., 2002 rpoS mutations and loss of general stress resistance in Escherichia coli populations as a consequence of conflict between competing stress responses. J. Bacteriol. 184: 806–811 11790751 10.1128/JB.184.3.806-811.2002 PMC139526

[bib20] Nyström T., 2004 MicroReview: Growth *vs.* maintenance: a trade-off dictated by RNA polymerase availability and sigma factor competition? Mol. Microbiol. 54: 855–862 15522072 10.1111/j.1365-2958.2004.04342.x

[bib21] Ochman H. Selander R. K., 1984 Standard reference strains of Escherichia coli from natural populations. J. Bacteriol. 157: 690–693 6363394 10.1128/jb.157.2.690-693.1984 PMC215307

[bib22] R Development Core Team, 2009 R: A Language and Environment for Statistical Computing. R Foundation for Statistical Computing, Vienna, Austria

[bib23] Robbe-Saule V. Algorta G. Rouilhac I. Norel F., 2003 Characterization of the RpoS status of clinical isolates of Salmonella enterica. Appl. Environ. Microbiol. 69: 4352–4358 12902215 10.1128/AEM.69.8.4352-4358.2003 PMC169149

[bib24] Spira B. de Almeida Toledo R. Maharjan R. P. Ferenci T., 2011 The uncertain consequences of transferring bacterial strains between laboratories - rpoS instability as an example. BMC Microbiol. 11: 248 22067413 10.1186/1471-2180-11-248 PMC3240573

[bib25] Stoebel D. M. Hokamp K. Last M. S. Dorman C. J., 2009 Compensatory evolution of gene regulation in response to stress by Escherichia coli lacking RpoS. PLoS Genet. 5: e1000671 19798444 10.1371/journal.pgen.1000671 PMC2744996

[bib26] Sutton A. Buencamino R. Eisenstark A., 2000 rpoS mutants in archival cultures of Salmonella enterica serovar typhimurium. J. Bacteriol. 182: 4375–4379 10913067 10.1128/jb.182.16.4375-4379.2000 PMC94605

[bib27] Tenaillon O. Skurnik D. Picard B. Denamur E., 2010 The population genetics of commensal Escherichia coli. Nat. Rev. Microbiol. 8: 207–217 20157339 10.1038/nrmicro2298

[bib28] Typas A. Becker G. Hengge R., 2007 The molecular basis of selective promoter activation by the sigmaS subunit of RNA polymerase. Mol. Microbiol. 63: 1296–1306 17302812 10.1111/j.1365-2958.2007.05601.x

[bib29] Walk S. T. Alm E. W. Gordon D. M. Ram J. L. Toranzos G. A. *et al*, 2009 Cryptic lineages of the genus Escherichia. Appl. Environ. Microbiol. 75: 6534–6544 19700542 10.1128/AEM.01262-09 PMC2765150

[bib30] White A. P. Sibley K. A. Sibley C. D. Wasmuth J. D. Schaefer R., 2011 Intergenic sequence comparison of Escherichia coli isolates reveals lifestyle adaptations but not host specificity. Appl. Environ. Microbiol. 77: 7620–7632 21908635 10.1128/AEM.05909-11 PMC3209187

[bib31] Zambrano M. M. Siegele D. A. Almirón M. Tormo A. Kolter R., 1993 Microbial competition: Escherichia coli mutants that take over stationary phase cultures. Science (New York, N.Y.) 259: 1757–1760 10.1126/science.7681219 7681219

