# Supplementary material for: Escherichia coli Lacking RpoS Are Rare in Natural Populations of Non-Pathogens
Source: G3 (Bethesda). 2012 Nov 1;2(11):1341–4. doi: 10.1534/g3.112.003855 (PMC3484664; doi:10.1534/g3.112.003855)
Supplement: Supporting Information [file supp_2_11_1341__index.html]

Supporting Information 

# *Escherichia coli* Lacking RpoS Are Rare in Natural Populations of Non-Pathogens

## Supporting Information for Snyder, Gordon, and Stoebel, 2012

**Files in this Data Supplement:**

- Table S1 - Strains used in this study (.txt, 4 KB)
